# Automated segmentation of vertebral cortex with 3D U-Net-based deep convolutional neural network

**DOI:** 10.3389/fbioe.2022.996723

**Published:** 2022-10-19

**Authors:** Yang Li, Qianqian Yao, Haitao Yu, Xiaofeng Xie, Zeren Shi, Shanshan Li, Hui Qiu, Changqin Li, Jian Qin

**Affiliations:** ^1^ Department of Radiology, The Second Affiliated Hospital of Shandong First Medical University, Tai’an, China; ^2^ Mechanical and Electrical Engineering College, Hainan University, Haikou, China; ^3^ Hangzhou Shimai Intelligent Technology Co., Ltd., Hangzhou, China

**Keywords:** cortical separation, 3D U-Net, deep learning, segmentation, artificial intelligence-AI

## Abstract

**Objectives:** We developed a 3D U-Net-based deep convolutional neural network for the automatic segmentation of the vertebral cortex. The purpose of this study was to evaluate the accuracy of the 3D U-Net deep learning model.

**Methods:** In this study, a fully automated vertebral cortical segmentation method with 3D U-Net was developed, and ten-fold cross-validation was employed. Through data augmentation, we obtained 1,672 3D images of chest CT scans. Segmentation was performed using a conventional image processing method and manually corrected by a senior radiologist to create the gold standard. To compare the segmentation performance, 3D U-Net, Res U-Net, Ki U-Net, and Seg Net were used to segment the vertebral cortex in CT images. The segmentation performance of 3D U-Net and the other three deep learning algorithms was evaluated using DSC, mIoU, MPA, and FPS.

**Results:** The DSC, mIoU, and MPA of 3D U-Net are better than the other three strategies, reaching 0.71 ± 0.03, 0.74 ± 0.08, and 0.83 ± 0.02, respectively, indicating promising automated segmentation results. The FPS is slightly lower than that of Seg Net (23.09 ± 1.26 vs. 30.42 ± 3.57).

**Conclusion:** Cortical bone can be effectively segmented based on 3D U-net.

## 1 Introduction

Osteoporosis has progressively gained public awareness as a prevalent bone disorder. It is a systemic bone disease characterized by decreased bone mass and microarchitectural degeneration, which leads to bone fragility and increased fracture risk (Cheng et al., 2020). The cortical bone accounts for a small volume in the spine, but it bears 45%–75% of the load (Rockoff et al., 1969), and cortical bone is more closely related to bone strength and is more susceptible to mechanical forces than cancellous bone (Ye et al., 2020), which point to cortical bone’s role in osteoporotic fractures. The cortical shell is crucial to the vertebral body’s capacity to bear loads. As age-related bone mass loss progresses, the load-bearing role of the cortical sell increases significantly, making the cortex more prone to fracture (Cao et al., 2001). Loss of cortical bone mass and increasing cortical porosity result from an unbalance in intracortical remodeling, which also leads to osteoporotic fractures (Shah et al., 2018). Both the pathogenesis of osteoporotic fractures and the treatment options for osteoporosis are influenced by cortical bone strength. A healthy vertebral body cortex not only maintains the stability of the vertebral body but also protects the spinal cord from injury (Wallace et al., 2017). Palepu et al. (2019) found that the thickness of the vertebral cortex varied circumferentially within a vertebra and that this variation was distinct for each level of the lumbar spine. As a result, having detailed reference data for each vertebral cortical thickness might help you comprehend bone quality better. 3D images can not only show the integrity of cortical bone but also visually display the thickness of cortical bone at different levels.

Accurate image segmentation is critical for cortical thickness measurement, regional image analysis, and surgical planning. However, accurate segmentation of cortical bone is difficult due to partial volume artifacts caused by the low spatial resolution; second, the boundary between cortical and trabecular compartments is blurred and difficult to distinguish; and third, adjacent images overlap, making cortical bone segmentation even more difficult. Manual segmentation can produce good results in clinical practice, but it comes with its disadvantages: it is time-consuming and involves inter-rater variability in practice. The automatic segmentation techniques, in contrast, are not only quicker but are also built on measures agreed upon by several raters, thus improving consistency and reducing inter-rater variability.

Due to the rapid development of deep neural networks, it has shown satisfactory performance in many medical images processing such as classification, segmentation, detection, localization, and registration (Chan et al., 2020; Fu et al., 2020; Kalmet et al., 2020; Krishnan et al., 2020; Yan et al., 2020; Hassanzadeh et al., 2021; Li et al., 2021; van Sloun et al., 2021; Li et al., 2022), and has been widely used in many fields (Zaharchuk et al., 2018; Jiang et al., 2020; Chandra et al., 2021). Numerous studies have shown that high-precision results that may be used in clinics can be achieved when segmenting medical images. Mastmeyer et al. (2006) suggested a hierarchical 3D technique for segmenting vertebral bodies that combines traditional morphological procedures with more complicated processes to allow for a rough segmentation of vertebral bodies. Huang et al. (2009) created a fully automatic vertebra recognition and segmentation system for spine MRI that includes three stages: AdaBoost-based vertebra detection, detection refinement by robust curve fitting, and vertebra segmentation *via* an iterative normalized cut approach. For automatic vertebra segmentation and identification, Lessmann et al. (2019) suggested an iterative full convolutional neural network combining deep learning with CT and MRI. Using a fully convolutional neural network to segment and label vertebrae one by one, regardless of the number of visible vertebrae, the method achieves great segmentation accuracy. Kolarik et al. (2019) discovered a high-resolution 3D Dense-U-Net network capable of segmenting the spine in the input image at native resolution and processing 3D image data. Although the foregoing research has proven that segmentation of the complete spine or single vertebrae can yield good results, few studies have focused on cortical segmentation of the vertebrae. Buie et al. (2007) proposed the dual threshold technique, which involved two required threshold inputs for extracting the cortex’s periosteal and endosteal surfaces. Some morphological parameters of segmentation measured by the proposed method were in good agreement with the gold standard, while others were in poor agreement. A structure-based algorithm for differentiating cortical and trabecular bone had been presented by Ang (Ang et al., 2020). Bone connected to the cortex within a spatially local threshold value is identified and segregated from the remaining bone using the thickness of the cortex as a seed value, however, the algorithm has only been tested on the long bones of four species. Zebaze et al. (2013) described an automated method for segmenting bone into its compact-appearing cortex, transitional zone, and trabecular compartment from background and bone, but it was mainly used to quantify cortical porosity. Li et al. (2013) offered an automated cortical bone segmentation system for MD-CT imaging of the distal tibia *in vivo*. Using a modified fuzzy distance transform and connectivity analysis, it made use of more contextual and topologic information from the bone. For the segmentation of cortical and trabecular bone, Valentinitsch et al. (2012) described a novel, totally automated, threshold-independent technique. The program selected textural features with high distinguishing power automatically and trained a classifier to distinguish between cortical and trabecular bone. This literature had only performed experiments on a small number of cadaveric bones and was insufficient to cover the high variability introduced by normal anatomy and/or pathological skeletal processes. Deep learning algorithms are more and more widely used in image processing and segmentation, but few people have applied them to the segmentation of cortical bone, especially the vertebral cortex. Our proposed accurate vertebral cortical image segmentation method will help to provide quantitative information for the diagnosis, treatment, and surgical planning.

Based on the above, our objective was to propose a 3D-UNet deep learning model to segment the vertebral cortex automatically. Then, the segmentation abilities of the proposed model are evaluated for the vertebral cortex.

## 2 Materials and methods

### 2.1 Datasets

From March to October 2020, a total of 316 consecutive patients who had undergone thoracic CT spinal imaging in our hospital were collected retrospectively in our study. The inclusion criteria of the research subject were as follows: 1) 20–37 years old; 2) 18.5–24.0 BMI (body mass index); 3) complete clinical and imaging data. Exclusion criteria: 1) osteoporosis; 2) vertebral lesions 3) endocrine diseases and other diseases that affect the thickness of cortical bone, such as diabetes, parathyroid disease, rheumatoid disease, etc.; 4) long-term use of bisphosphonates, glucocorticoids, estrogens, and other drugs; 5) incomplete imaging examination. According to the inclusion and exclusion criteria, a total of 105 patients were enrolled in this study (71 males, 34 females, age range 20–37 years). This study was reviewed and approved by the local ethics committee (2020-035). Written informed consent was not required in this retrospective study by the institutional requirements.

**FIGURE 1:**
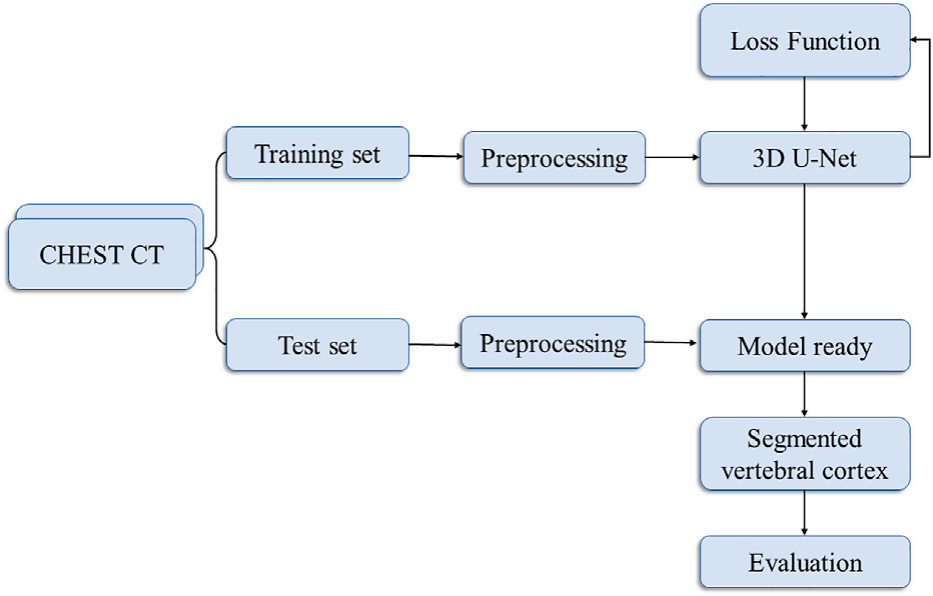
Flowchart of the proposed vertebral cortex segmentation method.

Our flowchart for vertebral cortex segmentation is shown in Figure 1. All the participants were performed on a 256-slice CT scanner (GE Healthcare), with image acquisition parameters listed in Table 1. The same protocol was used to process all of the scans.

**TABLE 1:** Image parameters of the dataset in our study.

Parameters	Description
Scan range	From T11 to L2
Tube voltage	120 kV
Tube current	335 mA
Layer thickness	1.25 mm
Layer spacing	1 mm
Image matrix	512 × 512
Window width	2000 HU
Window center	350 HU

**FIGURE 2:**
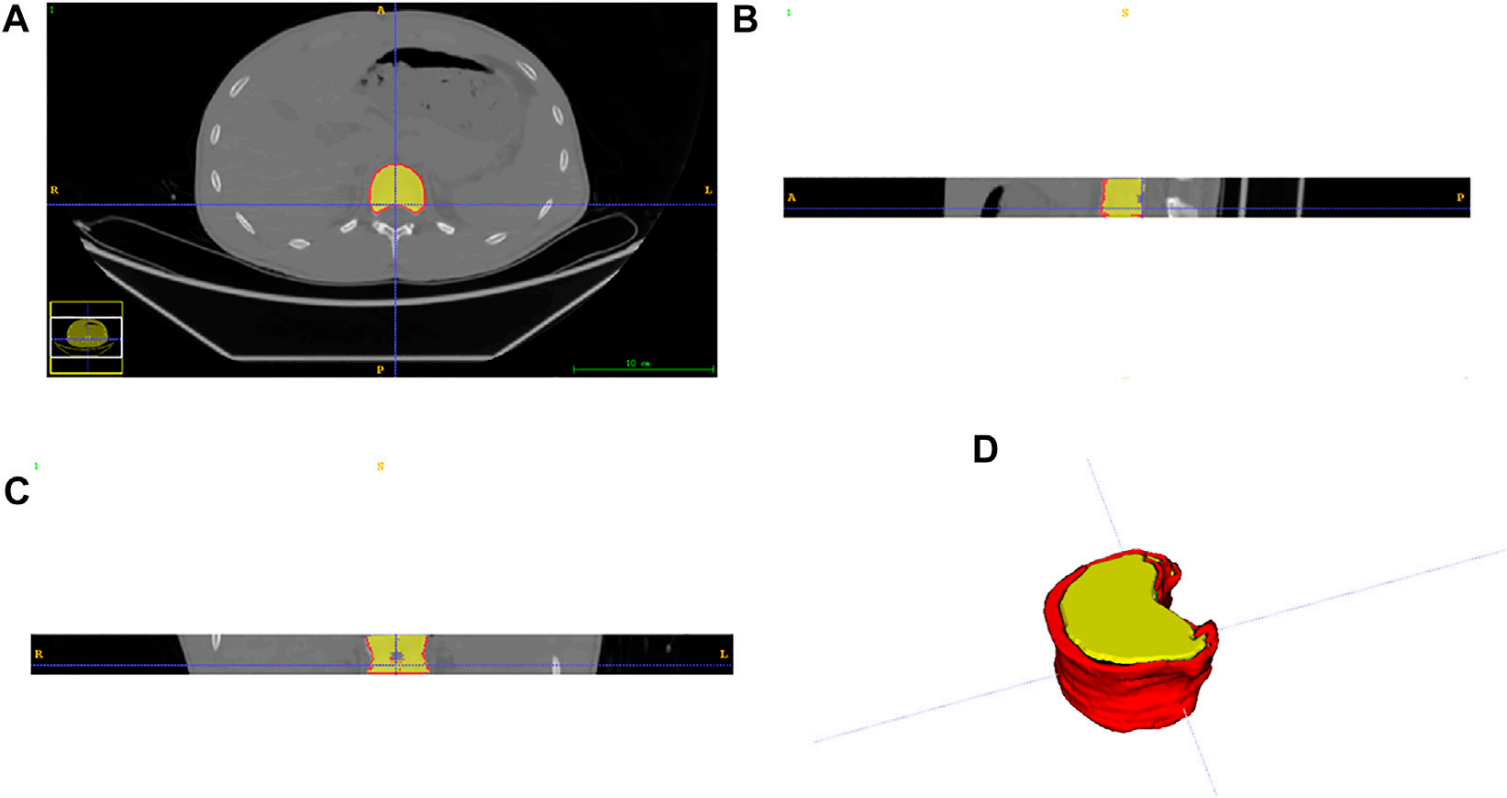
Three views and a 3D display of data. **(A)** axial plane; **(B)** coronal plane; **(C)** sagittal plane; **(D)** three-dimensional representation.

T11, T12, L1, and L2 were chosen from 105 participants who had CT scans. Each vertebral body was approximately 25–35 slices of axial images. The AW4.7 post-processing workstation (GE Healthcare) was used to perform vertical cross-section segmentation of the four vertebrae to obtain a more accurate and complete cortical morphology. A senior radiologist with more than 10 years of professional experience performed the delineation of the vertebral cortex with ITK-SNAP software, which was subsequently peer-reviewed by two other specialists. These vertebral cortex contours delineated by radiologists were referred to as the ground truth. Finally, both data and labels were converted into NIFTI format. Figure 2 shows the axial, coronal, sagittal, and 3D views of a single vertebral body, where the red area outside the vertebral body is the cortical bone we want to segment.

### 2.2 Data preprocessing

The image size is in the format (w, h, s), where w and h are the width and height of the image, respectively, w = h, s is the number of layers of the image, and s is between 30 and 40.

**FIGURE 3:**
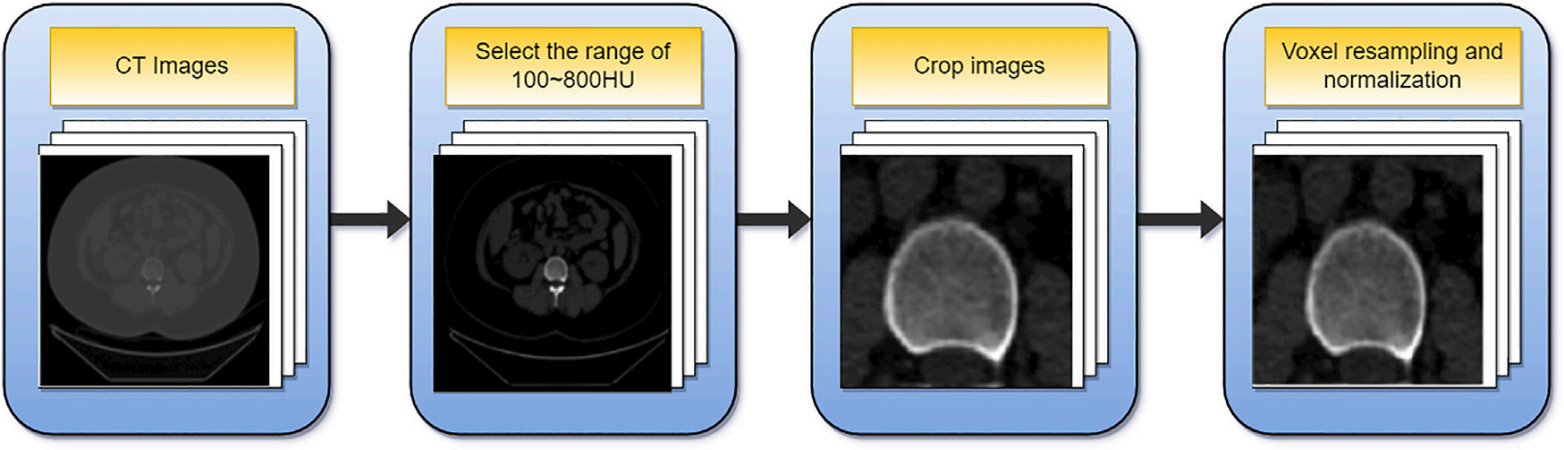
The flowchart of data preprocessing.

The data must be preprocessed due to the varying sizes of the images in the dataset and the limited fraction of cortical bone in the overall image. Figure 3 depicts the preprocessing flowchart. First, select the CT value range of 100–800 HU, then crop out the relevant area containing the cortical bone and resize the cropped image (256,256). In addition, since the voxel spacing of 3D images is not fixed, to facilitate subsequent processing, the voxel size is unified into (1, 1, 1) mm, and finally normalized. Supplementary Material gives a comprehensive explanation.

### 2.3 Fully automatic vertebral cortical segmentation based on 3D U-Net

#### 2.3.1 Automatic segmentation

**FIGURE 4:**
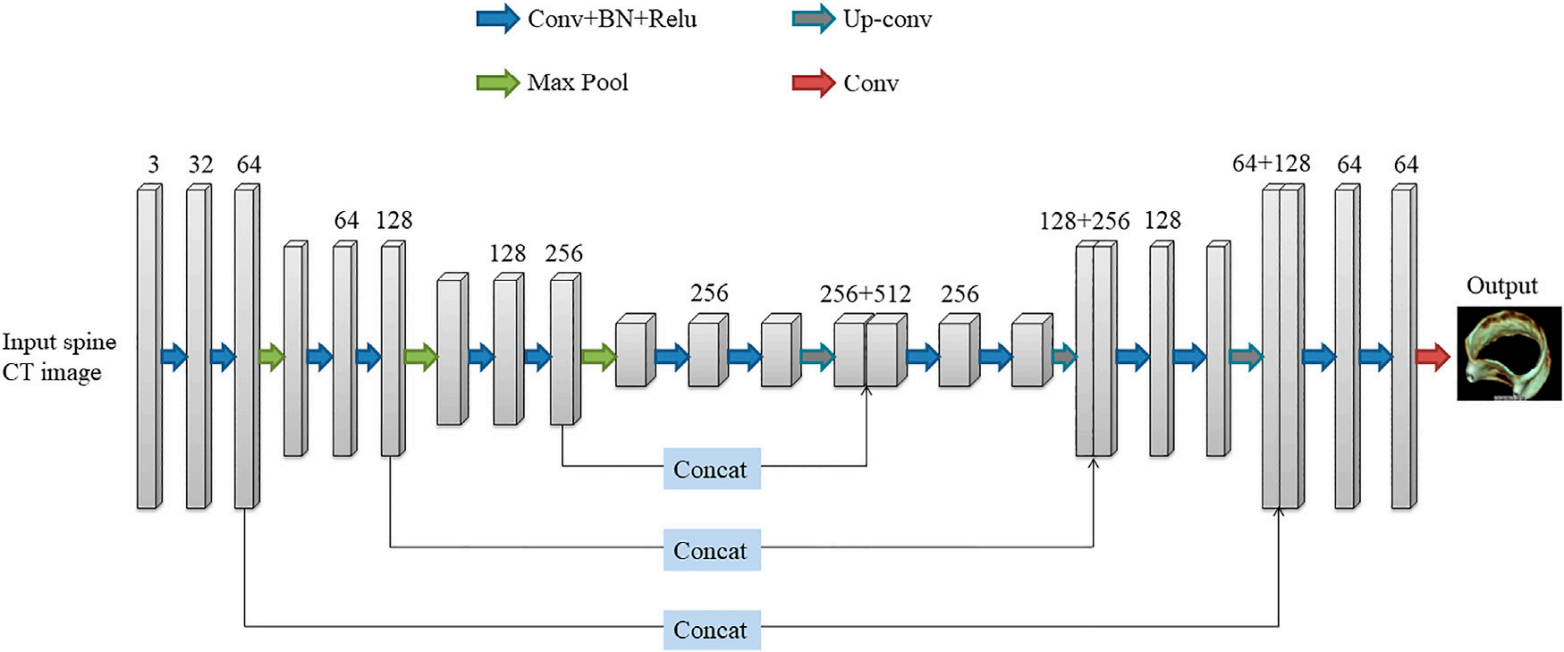
The architecture of 3D U-Net.

The deep learning network used in this paper is the 3D U-Net network proposed by Çiçek et al. (2016). The network was created to address the issue of medical image segmentation. U-Net (Ronneberger et al., 2015), which is a conventional “encoder-decoder” structure, is the foundation for the full 3D U-Net architecture. The encoder part is used to analyze the entire image and perform feature extraction and analysis, while the decoder creates a segmented block map (Figure 4). The entire 3D U-Net architecture is created based on U-Net, which is a typical “encoder-decoder” structure, the part of its change is to use 3D data in the processing process. 3D U-Net consists of the “encoder-decoder” structure. In the encoder structure, each layer of the neural network consists of two convolutions, each convolution followed by a batch normalization (BN) and a rectified linear unit (ReLU), and finally a 2 × 2×2 maximum pooling with strides of two in each dimension. In the decoder structure, each layer consists of a 2 × 2 × 2 up-convolution with strides of two in each dimension, followed by two 3 × 3 × 3 convolutions, each followed by a BN and a ReLU. At the same time, the result of the corresponding network layer on the encoder is used as part of the input of the decoder, so that the high-pixel feature information retained in the feature analysis can be collected, so that the image can be better synthesized.

#### 2.3.2 Loss function

A loss function is needed to quantify the error between the network output and the ground truth to evaluate the predictions and adjust the network parameters. In this study, we used three loss functions (DiceLoss, Weighted Cross-entropy, HybridLoss) to train and test the 3D U-Net network, and compared the effects of the three loss functions on the experimental results. The definition of DiceLoss, Weighted Cross-entropy, and HybridLoss are as follows:

DiceLoss:

$$DiceLoss\left(g,p\right)=1-\frac{2\overset{n}{\underset{i=1}{\sum }}p_{i}g_{i}}{\overset{n}{\underset{i=1}{\sum }}\left(p_{i}^{2}+g_{i}^{2}\right)}$$ (1)

*p* represents the predicted value; *g* represents the true value; *n* represents the number of voxel points in the 3D image. It can be seen from the formula that the background area will not be calculated for the loss, so the network focuses on the division of the cortex, which is conducive to the convergence of the network, and improves the segmentation accuracy.

Weighted Cross-entropy:

$$L_{WCE}=-\frac{1}{N}\overset{N}{\underset{i=1}{\sum }}\overset{C}{\underset{c=1}{\sum }}g_{i}^{c}\log s_{i}^{c}$$ (2)

In the above formula, N and C are the numbers of pixels and data categories respectively, g_i_ is the true label, and s_i_ is the predicted value.

Hybrid-Loss:

$$L_{HybridLoss}=-\frac{1}{N}\overset{N}{\underset{i=1}{\sum }}g_{i}\log s_{i}+\left(1-g_{i}\right)\log\left(1-s_{i}\right)+\frac{2\sum_{i=1}^{N}s_{i}g_{i}+\epsilon }{\sum_{i=1}^{N}s_{i}+\sum_{i=1}^{N}g_{i}+\epsilon }$$ (3)

Hybrid-Loss is the sum of the binary cross-entropy loss and the Dice loss, where N is the number of pixels, g_i_ is the true label, s_i_ is the predicted value, and ϵ is the smoothing factor to avoid the divisor being 0.

#### 2.3.3 Parameter setting

The original image was flipped horizontally and rotated 45 degrees clockwise and counterclockwise, and the data was expanded by 4 times. Three patch sizes were tested: (96, 96, 16), (128, 128, 16), (256, 256, 16). The network training epochs were set to 100, the Adam optimizer was selected, the initial learning rate was 0.0001, and the batch size was set to 6. Deep supervision was adopted to avoid the problem of gradient vanishing, and the initial value of the deep supervision attenuation coefficient was set to 0.4. During the training process, the learning rate and deep supervision coefficient decreased with the increase in training times, ensuring that the training tended to be stable.

#### 2.3.4 Device configuration

The experimental environment was as follows: CPU Intel (R) Core (TM) i7-9700F @ 3.00 GHz, GPU NVIDIA GeForce RTX 2070 SUPER, Windows10 operating system, Pytorch deep learning framework + python3.6.

### 2.4 Evaluation and statistical analysis

Each segmentation result was assessed and compared to the gold standard in this paper. We used four evaluation indicators, Dice Similarity Coefficient (DSC), mean Intersection over Union (mIoU), Mean Pixel Accuracy (MPA), and Frames Per Second (FPS), to evaluate the segmentation effect and inference speed of the model. Defined as follows:

$$DSC=\frac{2\left|P\cap T\right|}{\left|P\right|+\left|T\right|}$$ (4)

*P* is the set of predicted mask pixel locations, and *T* is the set of ground truth annotated pixel locations. *P∩T* represents the overlapping part of the two. The DSC value range from 1 to 0, with 1 being the best and 0 being the worst. The higher the value, the more accurate the segmentation is compared to the gold standard of radiologist segmentation.

$$mIoU=\frac{1}{k+1}\overset{k}{\underset{i=0}{\sum }}\frac{p_{ii}}{\overset{k}{\underset{j=0}{\sum }}pij+\overset{k}{\underset{j=0}{\sum }}p_{ji}-p_{ii}}$$ (5)

$$MPA=\frac{1}{k+1}\overset{k}{\underset{i=0}{\sum }}\frac{p_{ii}}{\overset{k}{\underset{j=0}{\sum }}p_{ij}}$$ (6)

*k* is the number of categories, *i* is the true value, *j* is the predicted value, and *p*_*i*j_ is the number of pixels that predict *i* as *j.*

$$FPS=\frac{1}{t}$$ (7)

*t* is the time taken by the model to infer an image, in seconds (s).

To better evaluate the segmentation accuracy of 3D U-Net, it is compared with Res U-Net (Xiao et al., 2018), Seg Net (Badrinarayanan et al., 2017), and Ki U-Net (Valanarasu et al., 2021) deep learning algorithms, respectively.

## 3 Result

### 3.1 Dataset

105 patients participated in the study, and a total of 420 thoracolumbar spine CT images were obtained. Among them, 2 images were excluded due to images being incomplete or images being of such low resolution for the radiologist to accurately annotate the ground truth. Finally, a total of 418 thoracolumbar spine CT images were obtained. The original images of 418 cases were horizontally flipped and rotated 45 degrees clockwise and counterclockwise for data enhancement, and 1,672 cases were obtained.

### 3.2 Patch size and loss function

In this experiment, three patch sizes and three loss functions were used for the 3D U-Net network respectively (Tables 2, 3), and Eq. 4 was used for evaluation. From Tables 2, 3, it can be seen that the optimum segmentation effect was achieved with a patch size of (256, 256, 16) using the DiceLoss function. This also proved that for the segmentation of small target areas, the training effect of sending the entire image to the network was better. At the same time, DiceLoss was more suitable for this experiment than other commonly used loss functions, and the segmentation effect was also the best.

**TABLE 2:** DSC values for different patch sizes and loss functions of the training set.

	(96, 96, 16)	(128, 128, 16)	(256, 256, 16)
DiceLoss	0.6576	0.6915	0.7240
Weighted cross-entropy	0.6678	0.6534	0.6803
HybridLoss	0.5778	0.5823	0.6176

**TABLE 3:** DSC values for different patch sizes and loss functions of the test set.

	(96, 96, 16)	(128, 128, 16)	(256, 256, 16)
DiceLoss	0.6635	0.6896	0.7128
Weighted cross-entropy	0.6554	0.6438	0.6733
HybridLoss	0.5622	0.5756	0.5978

### 3.3 Segmentation evaluation and comparison with other different algorithms

**FIGURE 5:**
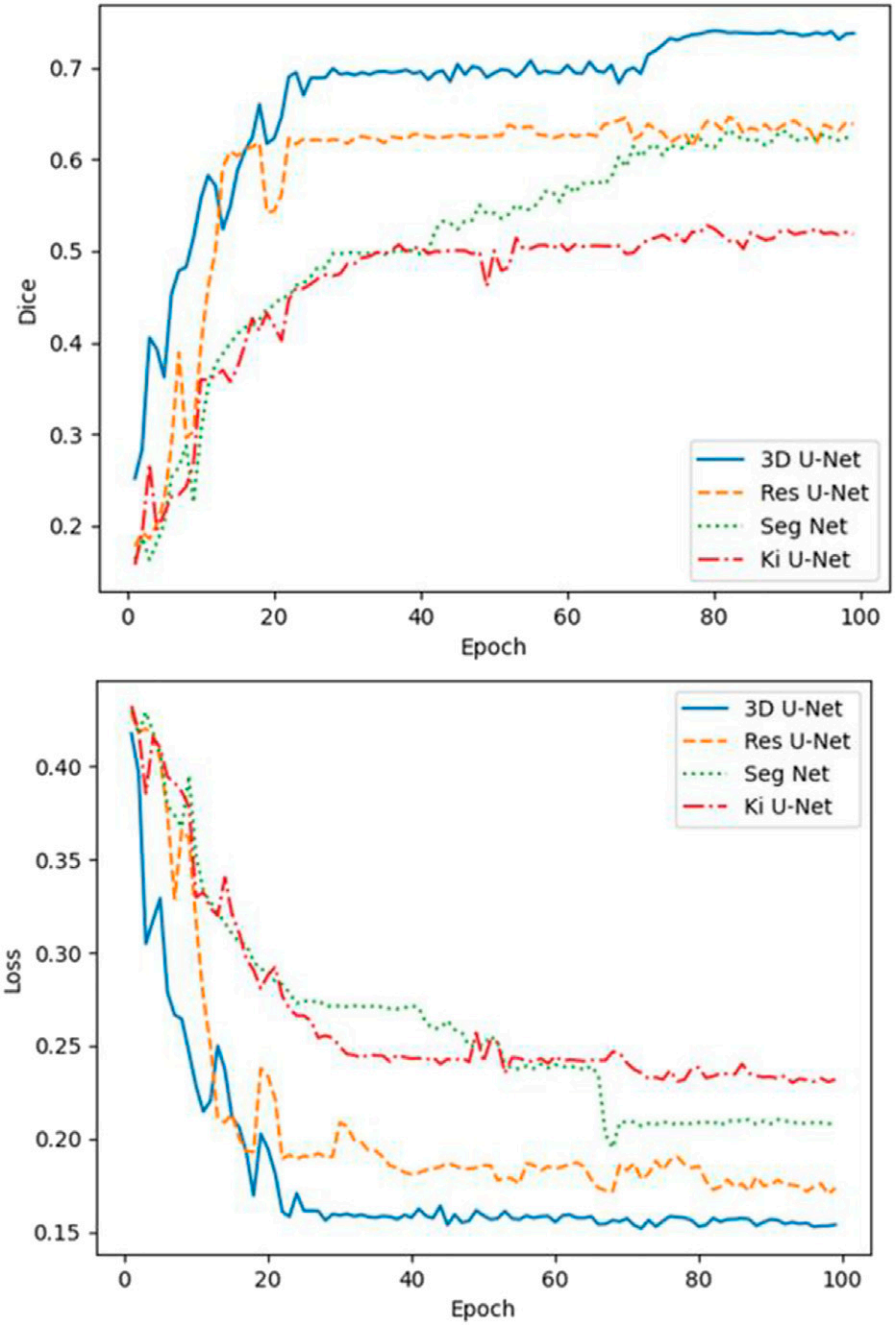
Loss and Dice values of 3D-Unet, Ki U-Net, Res U-Net, and Seg Net on the training set. The Loss value of the 3D-Unet algorithm is lower than the other three algorithms, while the Dice value is higher than the other three algorithms.

**FIGURE 6:**
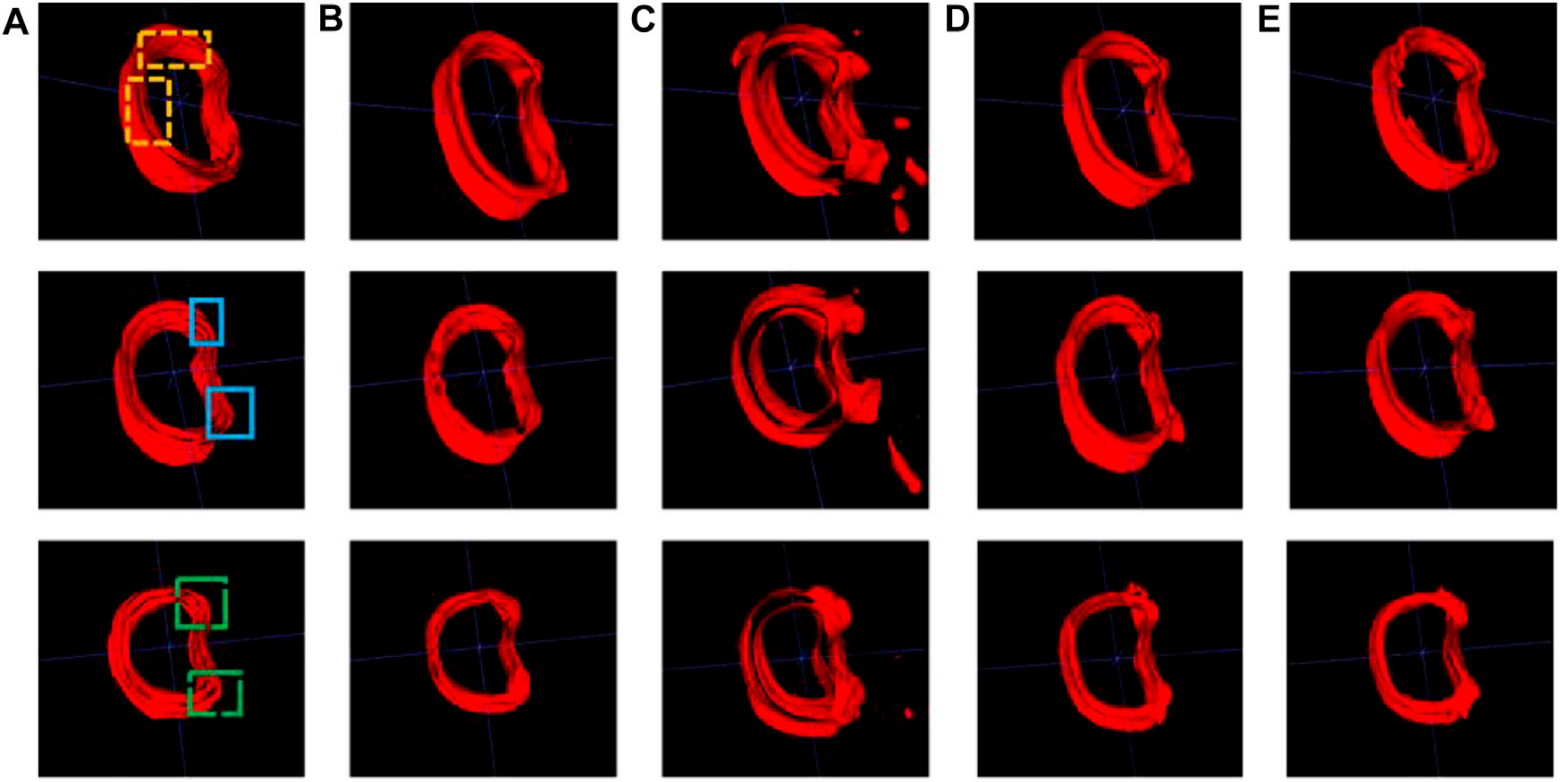
Cortical bone segmentation results (each row from left to right are the gold standard and segmentation results of the different algorithms). **(A)** Ground-truth; **(B)** 3D U-Net; **(C)** Ki U-Net; **(D)** Res U-Net; **(E)** Seg Net.

A comparative analysis was performed on the Res U-Net, Seg Net, Ki U-Net, and our proposed 3D U-Net. On the training set for 3D U-Net and the other three methods, we conducted a ten-fold cross-validation to determine which approach works the best. The test set was then tested using DSC, mIoU, MPA, and FPS (Tables 4, 5). It can be seen that the DSC, mIoU, and MPA of 3D U-Net were higher than those of the other three methods, indicating that the segmentation effect of this method is the best. The FPS is slightly lower than that of Seg Net, but since the main purpose of this experiment is not real-time, it is acceptable. Figure 5 represents the loss drop and Dice value of different algorithms on the training set, respectively. It can be seen from the above figures that the overall performance of the 3D U-Net algorithm is better than the other three methods, and the segmentation accuracy is higher. The Loss value of the 3D-Unet algorithm is lower than the other three algorithms, while the Dice value is higher than the other three algorithms. Figure 6 compares the difference between the ground truth and the segmentation results of 3D U-Net, Ki U-Net, Res U-Net, and Seg Net. The 3D U-Net segmentation effect is found to be the best, and the overall shape is the closest to the ground truth; Seg Net and Res U-Net will incorrectly segment surrounding tissue; The segmentation result of Ki U-Net is the worst, the shape is far from the gold standard and it will segment irrelevant tissues.

**TABLE 4:** DSC of the four methods on the training set.

Fold	3D U-Net	Res U-Net	Seg Net	Ki U-Net
1	0.6859	0.6457	0.5813	0.4981
2	0.7141	0.6381	0.5944	0.4732
3	0.7391	0.5964	0.618	0.5022
4	0.7438	0.6022	0.6154	0.5143
5	0.7412	0.5715	0.6321	0.4881
6	0.7223	0.6218	0.6092	0.4973
7	0.7501	0.6334	0.5873	0.5278
8	0.7349	0.6176	0.6239	0.5197
9	0.6934	0.6420	0.6158	0.5054
10	0.7155	0.6394	0.6245	0.4910
Mean	0.7240	0.6208	0.6102	0.5012

**TABLE 5:** Comparison of experimental results of different algorithms on the test set.

	DSC	mIoU	MPA	FPS
3D U-Net	0.71 ± 0.03	0.74 ± 0.08	0.83 ± 0.02	23.09 ± 1.26
Res U-Net	0.62 ± 0.07	0.66 ± 0.14	0.72 ± 0.05	20.33 ± 1.82
Seg Net	0.59 ± 0.06	0.61 ± 0.09	0.67 ± 0.04	30.42 ± 3.57
Ki U-Net	0.48 ± 0.07	0.52 ± 0.18	0.62 ± 0.06	20.96 ± 3.04

## 4 Discussion

In this study, we proposed a fully automated segmentation method based on 3D U-Net, which could obtain highly accurate results and reduce the workload of radiologists. The initial step in using finite element analysis, individualized surgical planning, and bone repair are to segment the cortical compartment. Manual segmentation is time-consuming and laborious, which is an obstacle to clinical application. Our skeletal functions are supported and protected by cortical bone, which is also vital in determining bone strength and fracture risk. This is the first time when 3D U-Net deep learning model was applied to vertebral cortex segmentation.

Since 3D images are very common in the medical field, most previous literature focused on 2D images (Malinda et al., 2019; Cho et al., 2020). If we want to train these models to solve tasks, we need to convert 3D images into 2D slices layer by layer for labeling. However, using 2D slice data to train a model is not only time-consuming and laborious but also may ignore the connection between layers. In addition, training with all the data of the entire 3D volume is inefficient and likely to cause overfitting, and it is impossible to create a large amount of data to optimize training. Therefore, based on the above discussion, the 3D U-Net was selected in this study, which was suitable for 3D medical images, and the experiment also showed that the network had a good segmentation effect on vertebral cortical bones.

At the beginning of this study, 3 different patch sizes and different loss functions were first considered. After testing, it was found that while dicing the data into little pieces might speed up the model convergence and reduce training time, it sacrifices some segmentation accuracy, therefore this study chose the patch size (256, 256, 16). In addition, compared with the Weighted Cross-entropy and HybridLoss loss functions, DiceLoss does not calculate the background area in the loss, that is, it focuses more on the segmentation of the cortical bone, which is more suitable for the task of this study. This experiment demonstrated that selecting the optimal patch size and loss function for a certain segmentation job can significantly increase the segmentation effect.

From the performance of the evaluation indicators listed in Table 3, it could be seen that the segmentation effect of 3D U-Net was superior to the other three methods. 3D U-Net had the advantage of utilizing the spatial information of 3D images, not ignoring the relationship between layers in the image, and using stitching procedures to merge low-level and high-level semantic features, allowing it to better restore the tissue’s structure and overall contours. In terms of inference speed, 3D U-Net was slightly lower than Seg Net, because the data used by 3D U-Net was three-dimensional, which consumed more computer memory than two-dimensional images. This outcome was acceptable because the segmentation job in this study was more concerned with accuracy than inference speed. The benefits of 3D U-Net resulted in a considerable improvement in segmentation accuracy, proving the method’s rationality in this study.

From Figure 6, we can see that for the 3 randomly selected test images, compared to the ground truth, Ki U-Net has the worst segmentation effect. It will not only incorrectly segment other tissues around the cortical bone, but also the overall shape is incomplete. The cortical bone region has obvious separation, which is far from the ground truth, and the segmentation performance of 3D U-Net is the closest. In the two yellow areas marked in the first image, it can be seen that the inner side of the cortical bone segmented by Res U-Net and Seg Net is relatively smooth, while the gold standard has obvious texture. At the same time, there are missing breaks in the segmentation of Seg Net, and the overall shape of the segmentation of 3D U-Net and Res U-Net is relatively complete. In the two blue regions marked in the second image, Res U-Net and Seg Net segment other surrounding tissues that do not belong to cortical bone, while 3D U-Net does not. In the two green areas marked in the third image, Res U-Net and Seg Net also have over-segmentation, and the middle area of Res U-Net segmentation is particularly obvious. Due to the fusion of cortical bone and cancellous bone or the incomplete shape of cortical bone in the adjacent layers of the 3D images in the dataset, the other three methods cannot achieve good segmentation results in this case. The input of the 3D U-Net is a three-dimensional image, which considers the connection between layers and makes better use of spatial information, so the best segmentation effect can be obtained.

To further improve the accuracy of cortical bone segmentation, there is still a lot of work to be done. First, modify the network structure to improve the segmentation ability for the fine structure. Second, further data collection is needed to increase the number of training samples. Third, since the cortical bone regions are difficult to capture and the manual calibration is time-consuming and laborious, consider using fewer and high-quality labels in combination with other methods to delineate the cortical bone.

## 5 Conclusion

In this work, we proposed a fully automated cortical bone segmentation method based on 3D U-Net. The obtained results were quantitatively evaluated on the entire dataset. Our proposed model not only achieved satisfactory segmentation accuracy, but it was also computationally efficient at inference time. Our results also showed that 3D U-Net significantly improved segmentation results compared to other deep learning algorithms.

## Data Availability

The raw data supporting the conclusion of this article will be made available by the authors, without undue reservation.
