# Supplementary material for: Automated segmentation of vertebral cortex with 3D U-Net-based deep convolutional neural network
Source: Front Bioeng Biotechnol. 2022 Oct 19;10:996723. doi: 10.3389/fbioe.2022.996723 (PMC9626964; doi:10.3389/fbioe.2022.996723)
Supplement: Supplementary file 1 [file Table1.DOCX]

**Supplementary Information**

**Data preprocessing**

1) Resampling

In medical images, the purpose of resampling is to normalize voxels of different sizes in a medical image to the same size, and a voxel is the smallest unit in a 3D medical image. An important reason why the convolution operation in the convolutional neural network is proposed is that similar blocks in the image can use shared convolution to extract features, so resampling all images can reduce the inconsistency between different images and facilitate convolution operations to extract common features. In this study, since the pixel spacing of the images in the dataset was inconsistent, a resampling operation was required. The new spacing is set to [1.0, 1.0, 1.0]. The specific operations were as follows: 1) Use SimpleITK to read data, and get the pixel spacing of the original image. 2) Divide the original pixel spacing of the image by the new pixel spacing to get the scaling factor, then multiply the original size of the image by this scaling factor to get the new image size. 3) Perform round processing on the size of the image, and divide the obtained value by the original size of the image to obtain a new scale factor. 4) Use the newly obtained scale coefficient to perform an interpolation operation.

2) Threshold cut-off normalization

Because CT images are different from natural images, the gray value range of natural images is generally 0 to 255, while different gray values in CT images correspond to different tissues and organs of the human body, ranging from -1024HU (all black) to 3071HU (all white). For the specific task of this study, the HU value is intercepted to the range of 100~800HU. The specific normalization operation is: 1) The minimum value (Min_HU_) = 100 and the maximum value (Max_HU_) =800. 2) (Image HU value-minimum value) / (maximum value-minimum value). 3) If the calculation result is greater than 1, it is set to 1, and if it is less than 0, it is set to 0, so as to achieve normalization.

3) Extracting only the slices containing cortical bone

When training a deep network, the more targeted the target area is, the better the result is. Therefore, in this study, only those slices containing cortical bone were extracted from the spine data set before training the model, and the processed data were used for training.
